# *Pannonibacter anstelovis* sp. nov. Isolated from Two Cases of Bloodstream Infections in Paediatric Patients

**DOI:** 10.3390/microorganisms12040799

**Published:** 2024-04-15

**Authors:** Stefano Castellana, Vittoriana De Laurentiis, Angelica Bianco, Laura Del Sambro, Massimo Grassi, Francesco De Leonardis, Anna Maria Derobertis, Carmen De Carlo, Eleonora Sparapano, Adriana Mosca, Stefania Stolfa, Luigi Ronga, Luigi Santacroce, Maria Chironna, Michela Parisi, Loredana Capozzi, Antonio Parisi

**Affiliations:** 1 Istituto Zooprofilattico Sperimentale della Puglia e della Basilicata, 71121 Foggia, Italy; stefano.castellana@izspb.it (S.C.); angelica.bianco@izspb.it (A.B.); laura.delsambro@izspb.it (L.D.S.); annamaria.derobertis@izspb.it (A.M.D.); antonio.parisi@izspb.it (A.P.); 2 UOC Microbiology and Virology, Azienda Ospedaliera-Universitaria Policlinico of Bari, 70124 Bari, Italy; vittoriana.delaurentiis@policlinico.ba.it (V.D.L.); carmela.decarlo@policlinico.ba.it (C.D.C.); eleonora.sparapano@policlinico.ba.it (E.S.); stefania.stolfa@policlinico.ba.it (S.S.); luigi.ronga@policlinico.ba.it (L.R.); 3 Division of Paediatric Haematology and Oncology, Azienda Ospedaliera-Universitaria Policlinico of Bari, 70124 Bari, Italy; massimo.grassi@policlinico.ba.it (M.G.); francesco.deleonardis@policlinico.ba.it (F.D.L.); 4 Department of Interdisciplinary Medicine, School of Medicine, University of Bari “Aldo Moro”, 70124 Bari, Italy; adriana.mosca@uniba.it (A.M.); luigi.santacroce@uniba.it (L.S.); 5 Department of Interdisciplinary Medicine, Hygiene Section, University of Bari “Aldo Moro”, 70124 Bari, Italy; maria.chironna@uniba.it; 6 University-Hospital Pediatric Department, Bambino Gesù Paediatric Hospital, 00165 Rome, Italy; michela.parisi@opbg.net

**Keywords:** Pannonibacter, environmental bacteria, whole-genome sequencing, putative novel species, hospital acquired infections

## Abstract

This study describes two cases of bacteraemia sustained by a new putative Pannonibacter species isolated at the U.O.C. of Microbiology and Virology of the Policlinico of Bari (Bari, Italy) from the blood cultures of two patients admitted to the Paediatric Oncohaematology Unit. Pannonibacter spp. is an environmental Gram-negative bacterium not commonly associated with nosocomial infections. Species identification was performed using Sanger sequencing of the 16S rRNA gene and Whole-Genome Sequencing (WGS) for both strains. Genomic analyses for the two isolates, BLAST similarity search, and phylogeny for the 16S rDNA sequences lead to an assignment to the species *Pannonibacter phragmitetus*. However, by performing ANIb, ANIm, tetranucleotide correlation, and DNA-DNA digital hybridization, analyses of the two draft genomes showed that they were very different from those of the species *P. phragmitetus*. MALDI-TOF analysis, assessment of antimicrobial susceptibility by E-test method, and Analytical Profile Index (API) tests were also performed. This result highlights how environmental bacterial species can easily adapt to the human host and, especially in nosocomial environments, also gain pathogenic potential through antimicrobial resistance.

## 1. Introduction

The genus Pannonibacter belongs to the Stappiaceae, a family included among the Alphaproteobacteria. To our knowledge, just three species, namely, *P. carbonis*, *P. indicus*, and *P. phragmitetus*, have been described and included in the genus Pannonibacter [1,2,3]. Interestingly, the taxon includes species that have been isolated in natural habitats, all associated with water sources and soil contact such as lakes, hot springs, and mine waters. Based on the information in the literature, it could be defined as an environmental bacterial genus. In recent years, however, there have been studies that have particularly associated the species *P. phragmitetus* with pathological processes in humans [4,5,6].

Very few cases of nosocomial infections sustained by not commonly HAIs-associated (“Hospital Acquired Infections”) bacteria are reported in the literature [7]. We assume that this phenomenon is underestimated, since classic HAIs-associated bacterial species are usually investigated in bacteraemia.

One of the major public health challenges of our century is the spread of bacterial resistance to antibiotics. To date, this trend is increasing globally and, especially in communitarian and nosocomial settings, for example, in Italy from 2019 to 2022, the prevalence of carbapenemase-resistant microorganisms (CPOs) increased from 2.62% to 4.56%, the percentage of MRSA increased from 1.84% to 2.81%, and the percentage of VREs increased from 0.58% to 2.21% [8,9,10]. The very wide spread of genetic determinants responsible for antibiotic resistance also makes the presence and entry of environmental bacteria into hospital settings of crucial importance. In fact, exposure to antibiotic molecules or other antimicrobial compounds [11] and sharing ecological niches with microorganisms that have resistance genes can potentially promote the evolution of resistant bacteria [12,13,14].

Between late July and early August 2022, two blood cultures from Pediatric Hematology and Oncology Unit were received at the Microbiology U.O.C. of the Policlinico of Bari. The blood culture samples were collected from two paediatric patients admitted to two different areas of the same ward.

Patient 1 (Pt1) was a 2-year-old male child affected by T-cell acute lymphoblastic leukaemia (LLA-T) that was admitted to the ward on 22 July 2022 with febrile neutropenia. On 9th August, following the onset of fever (38.5 °C), a blood culture was carried out, which tested positive after 24 h (time-to-positivity (TTP)).

Patient 2 (Pt2) was a 3-year-old male child with congenital pyelectasis of the left side, having been diagnosed with neuroblastoma in September 2020. On 23 July 2022, 7 days after admission and the start of intensive chemotherapy, Pt2 had a febrile peak and a blood culture was performed, which resulted positive after 32 h.

For both patients, seeding positive blood cultures allowed for the growth of only a Gram-negative bacterial strain. Preliminary analyses for species identification allowed for the classification of the two strains as *Pannonibacter phragmitetus*, but further molecular investigations provided the clue that this might represent a new bacterial species. Pannonibacter spp. are Gram-negative, facultative anaerobic, alkaline-tolerant, and environmental bacteria, which can occasionally cause infections to humans.

## 2. Materials and Methods

### 2.1. Sample Collection

Blood was collected according to informed consent signed by the patients’ respective parents. The two blood cultures, received from Pediatric Hematology and Oncology Unit on 23 July and 9 August 2022, respectively, were incubated in the BacT/ALERT^®^ 3D automated microbial detection system (biòMerieux, Marcy-l′Étoile, France), reporting a positive result for bottles in which bacterial or fungal growth was detected. From the positive blood cultures of Pt1 (24 h of TTP) and Pt2 (32 h of TTP), slides were prepared with a blood smear from the flask, which were then subjected to Gram staining in order to characterize the bacteria or fungi grown in the bottle. An aliquot of blood was taken sterilely through a needle from the flask and was then sown on different types of agar media, respectively, such as Chocolate Agar + PolyViteX TM (PVX) (incubated at 37 °C in 5% CO_2_ atmosphere), Colistin-Nalidixic Acid agar (CNA), MacConkey agar, Mannitol Salt agar (MSA), and Sabouraud dextrose agar with 0.5% chloramphenicol. The last four media agar were incubated at 37 °C. Subsequently, the bacterial colonies grown in culture were dissolved in a drop of 0.9% saline solution on a slide, and the slide was subjected to Gram staining and microscopic observation.

### 2.2. In Vitro Analysis

For both Gram-negative bacterial strains isolated from the blood cultures of Pt1 and Pt2, species identification was performed by MALDI-TOF (VITEK-MS, bioMérieux, Marcy-l′Étoile, France), and Minimum Inhibitory Concentration (MIC) was determined by performing the E-Test method at the Microbiology U.O.C. of the Policlinico of Bari (Bari, Italy). For antimicrobial susceptibility evaluation on Pannonibacter spp. isolates, we performed the E-Test method using Mueller Hinton II Agar (MH agar) produced by Liofilchem, a standardized medium for the antimicrobial susceptibility testing of common fast-growing aerobic microorganisms, as recommended by the European Committee for Antimicrobial Susceptibility Testing (EUCAST). Inoculum suspensions were prepared in 0.9% saline solution and adjusted to a final concentration of 0.5 McFarland according to the standard with approximately 1–5 × 10^6^ CFU/mL.

According to the manufacturer’s instructions, the bacterial suspension was directly seeded on the agar plates, and the agar surface was allowed to dry for 15 min before placing the E-test strips on it. Thereafter, the plate was incubated at 37 °C for 24 h. The formation of an elliptical halo around the E-test strip is indicative of the antimicrobial sensitivity of the isolate. The edges of the halo formed around the strip converged on a Minimum Inhibitory Concentration (MIC) value, and it was usually compared with standard EUCAST values to determine the sensitivity of the strain.

We tested by E-test strips of some antimicrobial molecules: Ceftolozane/Tazobactam (CT 0.016–256 µg/mL), Ceftazidime/Avibactam (CZA 0.016–256 µg/mL), Cefepime (FEP 0.016–256 µg/mL), Meropenem (MRP 0.016–256 µg/mL), and Piperacillin-Tazobactam (TZP 0.016–256 µg/mL).

Clinical breakpoints or interpretative criteria for the antimicrobial agents tested are not available for Pannonibacter spp. in the EUCAST documents.

Molecular Biology Laboratory of the Istituto Zooprofilattico Sperimentale della Puglia e della Basilicata (IZSPB) (Putignano, Bari, Italy) performed, for both strains, the Analytical Profile Index (API), allowing for a fast species identification based on biochemical tests, using API 20E (specific for Enterobacteriaceae and other non-fastidious, Gram- negative rods) and API 50 CHB (specific for Bacillus and related genera, as well as Gram-negative rods belonging to the Enterobacteriaceae and Vibrionaceae families) galleries. The biochemical test values were submitted to the APIWEB database.

### 2.3. 16S rDNA Sanger Sequencing

In order to assign a taxonomical classification, the Molecular Biology Laboratory of the IZSPB initially performed the Sanger sequencing of the 16S rRNA gene. Genomic DNA was extracted from isolated colonies of both strains using DNeasy Blood and Tissue Kit (Qiagen, Hilden, Germany), according to the manufacturer’s protocol.

The two isolates were preliminarily identified by PCR and Sanger sequencing of a partial 16S rDNA gene using the universal 16S rDNA primers 517F and 1541R [15]. The obtained amplicons were purified using the ExoI/FAST AP enzyme systems (Thermo Scientific, Waltham, MA, USA). Purified PCR products were sequenced using a BigDye 3.1 Ready reaction mix (Applied Biosystems, Waltham, MA, USA) and a 3130 Genetic Analyzer (Applied Biosystems) automated sequencer for electrophoresis. The sequences were imported and assembled by means of the BioEdit [16] v.7.0.5 software, and assembled sequences were used as input BLAST-nucleotide in GenBank (https://blast.ncbi.nlm.nih.gov/Blast, accessed on 8 September 2022) [17].

### 2.4. Whole-Genome Sequencing

Considering the uncertain results obtained with Sanger sequencing of the 16S rDNA gene, WGS sequencing of both investigated strains was also performed at the Molecular Biology Laboratory at the IZSPB.

An indexed genomic library for each isolate was prepared using the Illumina DNA Prep Sample Preparation Kit (Illumina, San Diego, CA, USA), as previously described [18]. A 2 × 250 paired-end sequencing run was performed on the Illumina MiSeq platform. In addition to Illumina runs, a third-generation sequencing method (TGS) was carried out, which allowed us to produce long reads. Therefore, genomic DNA extracted from Pt2 bacterial isolate was subjected to long-read sequencing (without pre-amplification and PCR-based enrichment) on a MinION MK1C device with R.9.4.1 flowcells using the ligation protocol with barcoding kits to multiplex up to 12 samples (barcode kits EXP-NBD104), as described in [19].

### 2.5. Bioinformatics Analyses

Primary bioinformatics analyses were performed following a customized Galaxy v23.0.1 workflow on the Galaxy Europe platform (https://usegalaxy.eu/, accessed on 22 May 2023). It includes read quality control through Fastp [20] 0.20.1, genomic assembly by SPAdes [21] v.3.12.0, gene annotation (CDS, tRNA, tmRNA, and rRNA) through Prokka [22] v1.14.6, and assembly quality check by Quast [23] v5.0.2. Default parameters were imposed on the abovementioned tools. BUSCO [24] version 5.4.6 (used reference database: “alphaproteobacteria_odb10”) and CheckM [25] v1.2.0 (reference database: “o__Rhizobiales”) were used for checking gene content completeness and contamination.

Marker-base phylogeny (16S rDNA) was carried out by extracting “16S ribosomal RNA” gene sequences from the two “Prokka.ffn” output files. After they were aligned by Blast2seq in order to check for complete identity, one of them was given as the input reference sequence for NCBI BLAST Nucleotide against the rRNA databases [26]. Retrieved sequences with >90% similarity were extracted, re-aligned through ClustalOmega [27] (https://www.ebi.ac.uk/Tools/msa/clustalo/, accessed on 23 May 2023) with our reference sequence, and provided to IQ-TREE [28] webserver multicore version 1.6.12 (http://iqtree.cibiv.univie.ac.at/, accessed on 23 May 2023) for phylogenetic model selection and subsequent phylogenetic analysis. A graphical phylogenetic tree was generated by iTOL [29].

Comparative genomic analyses were performed by considering the JSpecies [30] (https://jspecies.ribohost.com/jspeciesws/#analyse, accessed on 23 May 2023) and TYGS [31] (https://tygs.dsmz.de/user_requests/new, accessed on 23 May 2023) web applications. JSpecies was implemented to evaluate the possibility that the draft genomes belong to any known prokaryotic species by integrating calculations of the following: Average Nucleotide Identity by BLAST+ and MUMmer (“ANIb” [32], “ANIm” [33]), Tetra-nucleotide correlations (“Tetra [34]”) across a user-defined genome set; Tetra-nucleotide Correlation Search (“TCS”) against JSpecies genome database. A “Pannonibacter genome set” was built by searching the curated GTDB [35] genome portal (https://gtdb.ecogenomic.org/searches?s=al&q=pannonibacter, accessed on 23 May 2023). Then, we collected assembly FASTA sequences (together with basic genome information) and used them as input data for the above-mentioned classification tools, together with the patient-derived sequences. Details of public Pannonibacter genomes are given in Supplementary Table S2: for species attribution, the *P. phragmitetus* DSM 14782 genomic sequence (NCBI accession: GCF_000382365.1) was considered as a reference system.

The TYGS web server was used for the estimation of the in silico DNA-DNA hybridization (“dDDH”) between genomes under study and TYGS internal genomic records.

The computational screening of antibiotic resistance and virulence factor genes was achieved by using the ABRicate [36] v0.8.1 tool within the Galaxy ARIES [37] Platform (https://aries.iss.it/, accessed on 23 May 2023). Furthermore, the presence of plasmid was evaluated by implementing the PlasmidFinder [38] 2.1 database; version: 18 January 2023, https://cge.food.dtu.dk/services/PlasmidFinder/, accessed on 23 May 2023). The annotation of protein domains for Pannonibacter amino acid sequences was carried out through InterPRO [39] v 5.59-91.0 scan within the Galaxy EU platform, obtaining, among others, protein extended names, UNIPROT [40], InterPRO, and Gene Ontology [41] accessions.

The Pannonibacter strain isolated from Pt2 blood culture was submitted to long read sequencing by means of an ONT MinION device, with the purpose of discovering potential long-range complex genomic features and ameliorating the quality of the draft assembly. A primary analysis was managed through MinKNOW v22.05.8 and Guppy [42] 6.1.5 for base calling within the MK1C platform. Then, Fastq file quality inspection was performed by NanoPlot [43] v1.41, while the consequent assembly/annotation steps were run within the Galaxy.eu platform (release 23.1). Briefly, adapters were trimmed and low-quality reads were discarded by means of Nanofilt v.2.8 [43], and a first assembly round was performed by the Canu 2.1.1 assembler [44]. Canu-corrected reads were further assembled by the Flye v2.9.1 assembler [45] and refined through Medaka v. 1.7.2 [46]. Statistics for the polished FASTA assembly were computed by QUAST v5.2.0. The presence of contaminant sequences was checked by the CheckM tool.

First, the long-read-based assembly was used as an input within the Proksee [47] web application (www.proksee.ca, accessed on 29 November 2023) together with MiSeq-generated assemblies and a *P. phragmitetus* reference genome. The BLAST-integrated tool for comparative genomics was applied with default parameters. Secondarily, we scanned short- and long-read-based assemblies of the same isolate for the presence of high-complexity genomic features, like mobile genetic elements or prophages. Proksee-integrated tools VirSorter [48] v1.1.1 and Phigaro [49] v1.0.1 were implemented for this purpose.

Finally, short-read and long-read assemblies were submitted to ribosomal Multi-locus Sequence Typing [50] for species identification on the pubMLST [51] website (https://pubmlst.org/species-id, accessed on 29 November 2023).

## 3. Results

### 3.1. Isolation, Microscopic, and Biochemical Characterization

**Figure 1:**
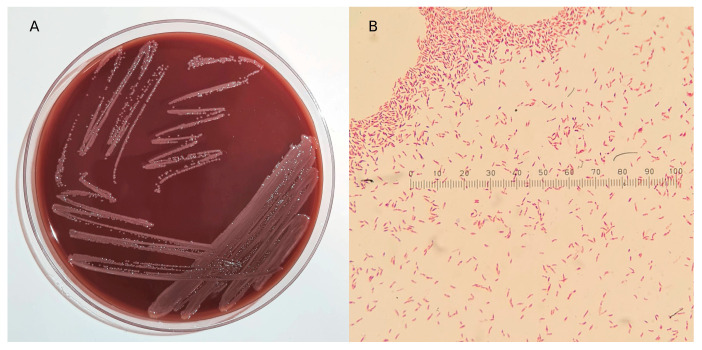
(**A**) Bacterial growth after seeding of blood culture bottles on PVX chocolate agar, detectable after 24 h incubation at 37 °C in 5% CO_2_ atmosphere. (**B**) Image acquired by optical microscope by observation at 100X of the slide on which a colony of Pannonibacter spp. was fixed and subjected to Gram staining.

The blood smear obtained from the two positive blood bottles, after Gram staining, allowed for the presence of Gram-negative bacilli to be detected by microscopic observation at 100X, among the red blood cells of the two patients. The seeding of the positive blood culture samples allowed for the growth on PVX agar after 24 h of incubation at 37 °C in microaerophilic conditions of milky-white mucous colonies (Figure 1A), characterized by an acrid odour. In a microscopic examination conducted by dissolving the colonies grown on PVX agar in a drop of saline, the bacteria appeared to be rod-shaped and Gram-negative (Figure 1B).

For both bacterial strains, MALDI-TOF analysis failed species identification several times. The antibiotic susceptibility, performed by E-test, revealed high MIC values for the two strains to aminoglycosides, third- and fourth-generation cephalosporins, and protected penicillin (Table 1).

Biochemical profiles obtained using API 20E and API 50 CHB kits for the two bacterial strains are shown in Table 2 and Table 3. The submission of these test values to the APIWEB database failed to identify the bacterial species, yielding an identity percentage of 49.3% with “Brevibacillus non-reactive” and 48.6% with “Bacillus non-reactive” for the Significant taxa. For the next taxon, an identity percentage of 1.9% was assigned with *Aneurinibacillus aneurinilyticus*. For the submission of values, only those with a marked colour variation (reported in Table 2 with the symbol “+”) were considered positive.

### 3.2. 16S Gene Analysis

The 16S rDNA gene sequences obtained by preliminary Sanger sequencing were 988 bp and 990 bp long (isolated from Pt1 and Pt2, respectively). They were both submitted to NCBI’s BLASTn tool, and the best similarity match was obtained for the 16S rDNA sequence of *Pannonibacter phragmitetus* DSM 14782 (NCBI Accession Number: MH507323.1) with 98% query coverage and 97.03% similarity, as well as a query coverage of 98% and a 97.23% similarity, respectively. Given the resistance profiles and the morphological characteristics of the isolates, we performed whole-genome analysis in order to better describe them.

### 3.3. Paired-End Sequencing Analysis

Illumina sequencing experiments for the reconstruction of two 4.6 Mbp long draft genomes were obtained, with N50 > 300 Kbp for each and a 62.58% GC content (details in Supplementary Table S1). The average depth of coverage was greater than 50X and 100X for Pt1 and Pt2 genomic sequences, respectively. The assemblies are deposited in NCBI Genbank (BioProject accession: PRJNA1073301), while the “Pt1” sequence (NCBI Genbank accession: GCA_036881715.1) can be considered as the reference genome for *P. anstelovis*.

A total of 49 tRNA, 3 rRNA, and 4150 protein-coding sequences were inferred along the 76–75 sequence contigs (Pt1 and Pt2, respectively), with 39% of the CDSs being annotated as hypothetical proteins (Supplementary Table S1). BUSCO analysis was used to assess a 100% completeness, i.e., structure and length of ortholog genes were in line with those within the closest lineage database (“*Alphaproteobacteria*”). No sequence contamination was detected by the CheckM tool, while a 99.4% completeness was estimated (taxonomical reference: *Rhizobiales* order).

**Figure 2:**
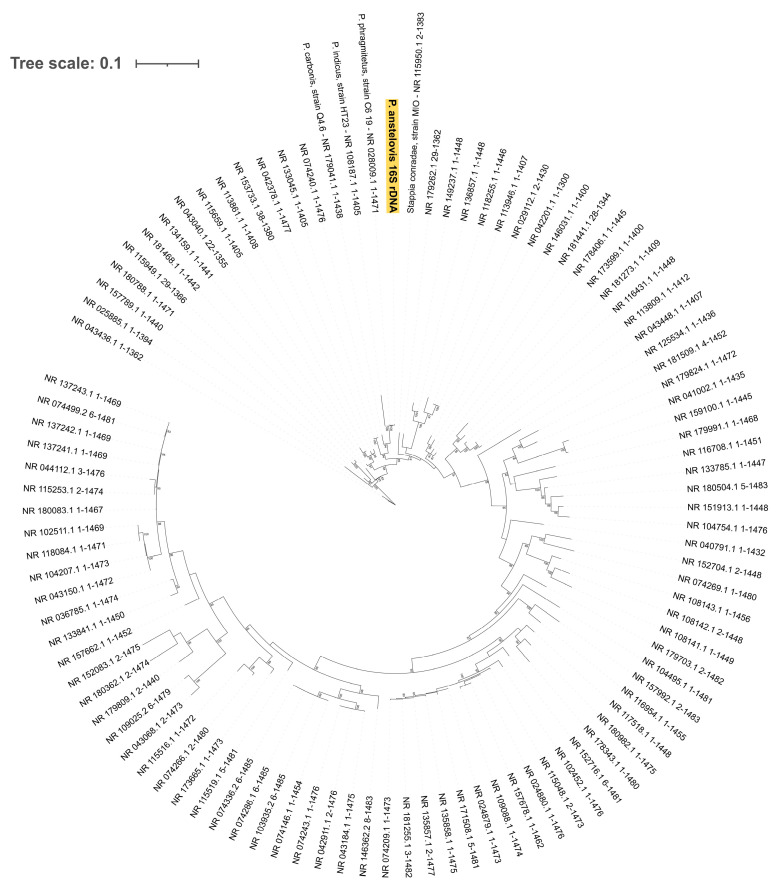
Phylogenetic tree for novel Pannonibacter 16S rDNA gene (yellow). Tips: NCBI accessions for rDNA genes from BLAST output. Positive numbers: branch support values as calculated by IQ-TREE web server.

As expected, 16S rDNA sequences from both samples were 100% identical. The BLASTn similarity search was then re-run: the draft Pannonibacter 16S ribosomal DNA revealed a 99.79% identity with *Pannonibacter phragmitetus* strain C6-19 16S ribosomal RNA, partial sequence (NCBI accession: NR_028009.1), and 98.29% with *Pannonibacter indicus* strain HT23 16S ribosomal RNA, partial sequence (NCBI accession: NR_108187.1). A phylogenetic analysis with IQ-TREE software 1.6.12 (substitution model: GTR + F + I + G4, selected by Bayesian Information Criterion score through IQ-TREE Model Selection module, bootstrap value for consensus tree generation: 1000) was in support of a taxonomical assignment as *Pannonibacter phragmitetus* strain C6-19 (Figure 2).

However, the predicted species nomenclature obtained for Pt1 (NCBI Genbank accession: GCA_036881715.1) and Pt2 genome sequences (NCBI Genbank accession: GCA_036881675.1) by rMLST analysis was *Pannonibacter phragmitetus* (support of 71% for both sequences), but the test found only 7 exact matches of the 53 genes encoding the bacterial ribosome protein subunits (rps genes).

In accordance with the rMLST data, genome-based classification methods applied to these two Pannonibacter isolates suggest a ′potential new species′ status.The BLAST-based Average Nucleotide Identity of Pannonibacter draft genomes against the 17 NCBI collected ones (details in Supplementary Table S2) was lower than the 95% species cutoff [32,52] for all pairwise comparisons (Supplementary Table S3); this was also confirmed by the Mummer similarity approach (Supplementary Table S4). A pairwise Tetra analysis returned a less definite scenario: tetra-nucleotide frequencies for both assembled genomes are below the 0.999 cutoff [53] (i.e., confident “same species” assignment) but in range (correlation > 0.989) with those associated to, especially, *Pannonibacter phragmitetus* and *Pannonibacter phragmitetus* DSM 14782 (NCBI accessions: GCF_900454465.1, GCF_000382365.1) (Supplementary Table S5). In addition to comparisons with our defined genome set, a further TETRA analysis through JSpecieS database search (“TCS” analysis) confirmed that tetra-nucleotide frequencies for newly assembled genomes are correlated with those related to *P. phragmitetus* and *P. indicus* strains. However, scores were not higher than the 0.999 cut-off but ranged from 0.991 to 0.996, meaning a possible “new species” status for the input assemblies (Supplementary Tables S6 and S7).

Analyses of digital DNA-DNA hybridization (“dDDH”) by means of the TYGS web server also evidenced a “potential new species” status for the two draft genomes. This was highlighted by considering the d4 [54] formula (more robust for incomplete/draft genomes with respect to d0 and d6). Both input genomes have d4 dDDH values below the species cutoff (dDDH > 70% [55]) with respect to the *Pannonibacter phragmitetus* DSM 14782 genome (around 44%) and the *Pannonibacter indicus* DSM 23407 one (around 41%) (Supplementary Table S8).

### 3.4. Oxford Nanopore Technology Re-Sequencing Analysis

The re-sequencing of the Pt2 isolate through the Oxford Nanopore Technology system produced a 4.65 Mbp-long genome (NCBI Genbank accession: GCA_036881735.1) consisting of two large contigs of 0.21 and 4.44 Mbp, with a slightly higher base composition with respect to short-read assemblies (62.78 vs. 62.58 GC%). A mean coverage greater than 50X was obtained starting from around 11,000 long reads. However, the genome completeness was lower than the one calculated for the short-read Pt2 assembly (89.3 vs. 99.4%, according CheckM tool), probably due to the error-prone long-read sequencing methodology. Further details for sequencing and assembly data are presented in Supplementary Table S12.

**Figure 3:**
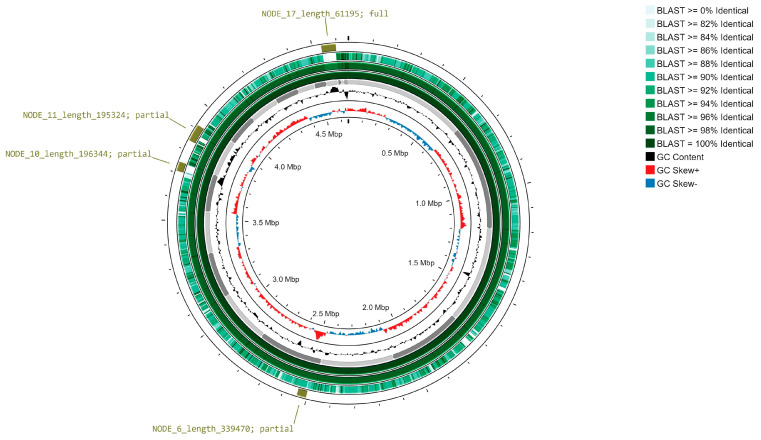
Proksee visualization of novel Pannonibacter genome assemblies and features. From inner to outer tracks: GC content skew among strand (plus/minus) for short−read assembled genome of novel Pannonibacter species (Pt2 version); corresponding global GC content; contig structures (light/dark grey) of short-read assembled genome; BLAST similarity percentage between the short-read assembled genomes from the two isolates (Pt2 vs. Pt1, green palette); BLAST similarity percentage between the short-read and long-read assembled genomes from one isolate (Pt2 vs. re-sequenced Pt2, green palette); BLAST similarity percentage between the short-read Pt2 assembled genome and *Pannonibacter phragmitetus* genome sequence (NCBI accession: GCA_000382365.1_ASM38236v1); prophage regions, as detected by VirSorter (labels indicate contig node and full/partial prediction).

The quality assessment of the three generated draft genomes was conducted through the ANIb similarity tool within the JSpecies web portal (accessed on 15 November 2023). The pairwise similarity results are summarized in Table 4 and Figure 3: *Pannonibacter anstelovis* sequences were >99% similar to each other, while ANI decreased toward 91% when compared to the *P. phragmitetus* reference sequence.

No evidence of plasmid was found on the Pt2 secondary genome version, while long-range complex genomic structures were detected along the three versions of the *Pannonibacter anstelovis* genome. In detail, three partial and one full prophage-associated regions (Figure 3) along the novel Pannonibacter spp. genome were classified within the *Siphoviridae* (full prophage annotated along assembly node 17, two partial sequences in nodes 6 and 10) and *Myoviridae* families (partial sequence within node 10). Details of the putative viral sequences are shown in Supplementary Table S13 (genomic positions are relative to the Pt 1 *P. anstelovis* assembly).

Given the high MIC values obtained in vitro to Amikacin, Cefepime, Cefotaxime, Ceftadizime, Gentamycin, Piperacillin/Tazobactam, and Tobramycin molecules, an initial prediction of resistance genes (“ARGs”) along Pannonibacter “Pt1” and “Pt2” genomes was carried out through ABRicate v 0.8.1 software, giving no evidence for the presence of known ARGs or virulence factor genes (Supplementary Table S9). Indeed, only partially covered (with a low sequence similarity) matching genes were found, with no biological connection to the tested drugs. Thus, the analysis was enlarged to the full Pannonibacter genomes, as scanned by Prokka and InterPRO applications (Supplementary Table S10). A manual inspection of the Pannonibacter proteome annotations evidenced that around 56 different genes would have a supposed antibiotic role against the tested drugs (Table 5, details in Supplementary Table S11).

For example, the genomes of the two sequences encode for several proteins of the beta-lactamase superfamily and several domains of penicillin-binding proteins (PBPs), which confer resistance to penicillin and cephalosporins. The InterPRO analysis also revealed the presence of sequences coding for proteins involved in specific resistance to tetracyclines, aminoglycosides, chloramphenicol, and Fosfomycin, as well as proteins that confer multi-resistance, such as efflux pumps and transporters. No significantly biased localization of ARGs was detected along the genomic sequences, nor in prophagic regions.The complete results of the functional annotation conducted using InterPRO on the *P. anstelovis* proteome are shown in Supplementary Table S10. Although, out of more than 4000 predicted proteins, there are some functional domains associated with flagellar proteins and lysis enzymes or secretion systems, there is no evidence of full-length and known virulence factors.

## 4. Discussion

The spread of environmental bacteria in hospital wards represents a health hazard for patients, particularly if they are immunocompromised or paediatric, as strains can gain significant antibiotic resistance. This study originated from an attempt to further investigate the isolation of two Gram-negative bacterial strains from the blood cultures of two different patients admitted to the same unit, whose species could not be established by conventional methods, such as MALDI-TOF and VITEK^®^ 2 GN ID card (Biomérieux). Molecular genotyping techniques were the only ones available to address the need to provide a diagnosis, such that therapeutic treatment could be set up for the two young oncohematology patients. It would probably be correct to investigate in this way whenever a difficult-to-identify strain is isolated from a clinical sample, especially in the case of samples such as blood or cerebro-spinal fluid.

Whole-Genome Shotgun has proven to be of great help in surveillance and monitoring the spread of emerging bacterial species and characterizing their potential antibiotic resistance. Indeed, in this study, it enabled the identification of a new putative bacterial species that would also circulate in hospital environments. Bacterial colonies with these growth characteristics (odour, colony shape, and colour), and which are difficult to identify using the standardised MALDI-TOF system, have never been detected in the microbiology laboratory of the Policlinico di Bari, either before, during these isolation phases, or after this finding. Indeed, genomic analyses for the two isolates likely support the idea that they cannot definitely be assigned to one of the known Pannonibacter species. Initially, BLAST similarity search and phylogenesis for the 16S rDNA sequences would support the assignment to *Pannonibacter phragmitetus* species, although the performance of such gene marker in species assignment cannot be fully conclusive [56]. Furthermore, it was decided to investigate the bacterial species and go beyond 16S rRNA Sanger sequencing, as we appreciated the in vitro antibiotic resistances exhibited by the strains. In fact, considering that these were bacterial strains isolated from the bloodstream of two young immunocompromised patients, following the outcome of 16S sequencing, the question arose as to how an environmental strain—such as *Pannonibacter phragmitetus*—could have those high MIC values in vitro. Hence, genomic sequencing of the two strains was carried out both to characterise the in silico resistome and to confirm species identification. In the literature [53,56], there have been explorations of how the reliability of the result for species identification conducted by WGS is superior to that obtained by sequencing a portion of 16S rRNA. Another consideration that prompted the use of WGS was the ability to investigate the genetic identity of two bacterial strains isolated 17 days apart in the same hospital ward.

Thus, after the Whole-Genome Sequencing of the two bacterial strains using Illumina and Nanopore technologies, a polyphasic approach was applied by performing ANIb, ANIm, tetranucleotide correlation, and DNA-DNA digital hybridization analyses. Most of them highlight how the two draft genomes are quite different from *P. phragmitetus* species. All tests performed on the whole-genome sequences of both bacterial strains yielded results that support the hypothesis that we are dealing with a new species of Pannonibacter. Specifically, the rMLST analysis was able to match only 7 ribosomal loci out of a total of 53 with those belonging to *Pannonibacter phragmitetus*, so 86.80% of the loci under analysis could not be matched to any bacterial species. The results obtained from the tests conducted with JSpecies and TYGS allow us to conclude that it represents a potential new species. However, in silico approaches to species determination greatly depends on sequencing yield, quality, and bioinformatics procedures. In this work, highly similar and consistent outcomes were obtained from the short-read sequencing experiments, while a lower-quality draft genome was obtained by using the ONT system. The annotation of genes throughout the long-read assembly was problematic, with an excessive prediction of short-length CDSs. Furthermore, a hybrid genome assembly approach was attempted, with poor qualitative results. Nonetheless, computational strategies for limiting the high error rate of third-generation sequencing platforms and ameliorating read assembly and alignment have been developed [57,58,59]. The usage of some of these error-correction and assembly refinement strategies allowed us to infer long-range genomic features along the Pannonibacter genome, such as prophage regions.. Improvements in sequencing chemistries, base calling algorithms, and combined assembly/alignment procedures will certainly increase the accuracy of long-read sequencing, making it suitable for sequence typing and genome functional annotation.

It should be pointed out that, unfortunately, the genus Pannonibacter has not been extensively studied and characterized, especially from a genetic perspective. To date, there has just ben 17 assemblies belonging to Pannonibacter spp. on the GTDB-curated database (Supplementary Table S2). This finding certainly represents a limitation for the purposes of species identification, genotyping analysis, and characterization of genetic determinants involved in antimicrobial resistance mechanisms. The optimal requirement for the definition of a new bacterial species would be to isolate several closely matched bacterial strains from different epidemiological backgrounds. However, we were able to isolate two genetically related strains from the bloodstream of two paediatric patients admitted to two different floors of the same department, almost twenty days apart. Once the microbiological outcome was obtained, both young patients were treated with broad-spectrum antibiotic therapy (Cefepime, Amikacin, and Teicoplanin), since no guidelines were available for Pannonibacter spp. for the interpretation of the breakpoints obtained with in vitro antimicrobial susceptibility assay tests. A subsequent gradual clinical improvement was recorded with antibiotic therapy until a normal body temperature was reached. Four days after the start of therapy, a new blood culture was performed for both patients, which, after 5 days of incubation, showed a negative result for the growth of microorganisms.

Although we did not isolate the same strain from other epidemiological settings, this finding underscore how easily an environmental bacterium can circulate within hospitals, managing to enter the bloodstream of patients who have not had direct contact.

A bloodstream infection caused by Pannonibacter spp., as well as by other bacteria, could account for the recorded febrile spike and pose a real risk to the health of seriously immunocompromised patients. In detail, we were not able to conduct functional assays on the hypothetical invasive capacity and virulence of *P. anstelovis* in addition to the assay of haemolysis capacity in vitro. By seeding the two isolated strains on blood agar, they showed no haemolysis. Thus, we had no sufficient in vitro and in silico data regarding virulence factors in these bacteria.

In addition, the blood smear slide taken immediately after the blood culture bottles tested positive allowed for the observation of the presence of morphologically identical Gram-negative bacilli. Microbiological culture then allowed for the growth of a single Gram-negative bacterial strain, and these results led to the association of the febrile spike with the presence of Pannonibacter spp. in the bloodstream and to the exclusion of the presence of other bacteria, including VBNC (viable but not culturable) strains that would have been visible by microscopy.

Analyses performed by classical screening systems for antibiotic resistance genes have failed due to the absence of genes referable to Pannonibacter species in the reference databases. Nevertheless, tests conducted directly on the amino acid sequences revealed portions coding for several proteins involved in resistance mechanisms. Analysing the sequenced bacterial genomes, it is only possible to provide a list of candidate genes that could be related to the resistances detected in vitro. This finding in particular applies to the molecules Cefepime, Cefotaxime, Ceftazidime, Piperacillin, and Amikacin. Sensitivities to Tetracyclines, Chloramphenicol, and generic multi-resistances found by genetic information were not tested in vitro.

The results obtained from this study certainly put a spotlight on the real risk represented by the ability of environmental bacteria to acquire major antimicrobial resistance. Especially for nosocomial settings, this represents a real hazard that needs to be monitored epidemiologically, as immunocompromised people could suffer serious infections from bacteria that are difficult to treat with therapies.

## Figures and Tables

**Table 1:** MIC values obtained for the antimicrobial molecules shown in the table, respectively, and obtained by seeding the bacterial colonies on MH medium and incubation for 24 °C at 37 °C.

Antimicrobial Molecule	MIC Pt 1 Strain	MIC Pt 2 Strain
Amikacin	>32	>32
Cefepime	16	16
Cefotaxime	16	16
Ceftazidime	32	32
Ciprofloxacin	0.5	0.5
Gentamycin	>8	>8
Imipenem	0.5	0.5
Meropenem	1	1
Piperacillin/Tazobactam	16	16
Tobramycin	>8	>8

**Table 2:** Biochemical profile of the API 50 CHB V4.1 Kit of the two Pannonibacter spp. isolates. Characters are scored as: +, positive; −, negative; W, weakly positive.

Well	Biochemical Tests	Results
0	Control	−
1	Glycerol	−
2	Erythritol	−
3	D-Arabinose	W
4	L-Arabinose	+
5	D-Ribose	−
6	D-Xylose	+
7	L-Xylose	W
8	D-Adonitol	−
9	Methyl-βD-Xylopyranoside	−
10	D-Galactose	W
11	D-Glucose	W
12	D-Fructose	W
13	D-Mannose	−
14	L-Sorbose	−
15	L-Rhamnose	−
16	Dulcitol	−
17	Inositol	−
18	D-Mannitol	−
19	D-Sorbitol	−
20	Methyl-αD-Mannopyranoside	−
21	Methyl-αD-Glucopyranoside	−
22	N-AcetylGlucosamine	−
23	Amygdalin	−
24	Arbutin	−
25	Esculin ferric citrate	+
26	Salicin	−
27	D-Cellobiose	W
28	D-Maltose	−
29	D-Lactose	−
30	D-Melibiose	−
31	D-Saccharose (sucrose)	−
32	D-Trehalose	−
33	Inulin	−
34	D-Melezitose	−
35	D-Raffinose	−
36	Amidon (starch)	−
37	Glycogen	−
38	Xylitol	−
39	Gentiobiose	−
40	D-Turanose	−
41	D-Lyxose	−
42	D-Tagatose	−
43	D-fucose	+
44	L-fucose	W
45	D-Arabitol	−
46	L-Arabitol	−
47	Potassium Gluconate	−
48	Potassium 2-KetoGluconate	−
49	Potassium 5-KetoGluconate	−

**Table 3:** Biochemical profile of the API 20 E Kit of the two Pannonibacter spp. isolates. Characters are scored as: +, positive; −, negative.

Well	Biochemical Tests	Results
1	ONPG	+
2	ADH	−
3	LDC	−
4	ODC	−
5	Citrate	+
6	Hydrogen sulphide	−
7	Urease	−
8	TDA	−
9	Indole	−
10	Voges-Proskauer	−
11	Gelatin	−
12	Nitrate	−

**Table 4:** Average Nucleotide Identity BLAST results for the three Pannonibacter assemblies (two isolates, one re-sequenced) and the *P. phragmitetus* reference sequence (most similar published genome). Percentage of aligned sites are shown in brackets.

Sample	Pt1 (MiSeq)	Pt2 (MiSeq)	Pt2 (ONT)	*Pannonibacter phragmitetus* DSM 14782 (GCA_000382365.1)
Pt1 (MiSeq)	-	99.99 (99.71)	99.76 (99.61)	91.21 (85.76)
Pt2(MiSeq)	100.00 (99.81)	-	99.77 (99.65)	91.23 (85.58)
Pt2-lr (ONT)	99.78 (99.61)	99.78 (99.67)	-	91.16 (85.67)
*Pannonibacter phragmitetus* DSM 14782 (GCA_000382365.1)	91.16 (83.33)	91.16 (83.26)	91.08 (83.29)	-

**Table 5:** Putative ARGs in *Pannonibacter anstelovis* genome: column 1, protein-coding gene accession; column 2: InterPRO amino acid domains/signatures with a putative role in antibiotic resistance; column 3: antimicrobial molecules against which the protein would likely have interactions.

Protein Accession	InterPRO Retained Domain	Antimicrobial Putative Interaction
FGIJAEJN_03410	Penicillin-binding protein, dimerisation domain	Cefepime, Cefotaxime, Ceftazidime, Piperacillin
FGIJAEJN_01305, FGIJAEJN_01921	Penicillin-binding protein, transpeptidase	Cefepime, Cefotaxime, Ceftazidime, Piperacillin
FGIJAEJN_02309, FGIJAEJN_03733, FGIJAEJN_01441, FGIJAEJN_01366	Beta-lactamase/transpeptidase-like	Cefepime, Cefotaxime, Ceftazidime, Piperacillin
FGIJAEJN_02847	Penicillin-binding protein transglycosylase domain	Cefepime, Cefotaxime, Ceftazidime, Piperacillin
FGIJAEJN_00520	Penicillin-binding protein, OB-like domain	Cefepime, Ceftazidime
FGIJAEJN_00222	Penicillin-binding protein, C-terminal domain superfamily	Piperacillin
FGIJAEJN_03193, FGIJAEJN_01113, FGIJAEJN_03499, FGIJAEJN_02312, FGIJAEJN_02878, FGIJAEJN_00760	Aminoglycoside phosphotransferase	Aminoglycoside
FGIJAEJN_00577, FGIJAEJN_02821, FGIJAEJN_02760, FGIJAEJN_03969, FGIJAEJN_00577, FGIJAEJN_03969	Drug resistance transporter Bcr/CmlA subfamily	Bicyclomycin, Chloramphenicol, Florfenicol
FGIJAEJN_01136, FGIJAEJN_00166	Chloramphenicol acetyltransferase-like domain superfamily	Chloramphenicol
FGIJAEJN_02702, FGIJAEJN_03166, FGIJAEJN_01924	Glyoxalase/fosfomycin resistance/dioxygenase domain	Fosfomycin
FGIJAEJN_02566	Multi antimicrobial extrusion protein	Multidrug
FGIJAEJN_00274	Multiple antibiotic resistance (MarC)-related	Multidrug
FGIJAEJN_00230	Drug resistance transporter EmrB-like	Multidrug
FGIJAEJN_00589	Small drug/metabolite transporter protein family	Multidrug
FGIJAEJN_03037, FGIJAEJN_00275, FGIJAEJN_01585, FGIJAEJN_04086, FGIJAEJN_03816, FGIJAEJN_00630	Multidrug efflux transporter AcrB TolC docking domain, DN/DC subdomains	Multidrug
FGIJAEJN_00589	Small multidrug resistance protein	Multidrug
FGIJAEJN_04073	Peptidase M74, penicillin-insensitive murein endopeptidase	Penicillin
FGIJAEJN_02114	Penicillin-binding, C-terminal	Penicillin
FGIJAEJN_00838	PBP domain	Penicillin
FGIJAEJN_02207	Beta-lactamase-related	Penicillin, Cephamycin, Cephalosporin
FGIJAEJN_01279	AmpG-like permease/Acetyl-coenzyme A transporter 1	Penicillin, Cephamycin, Cephalosporin
FGIJAEJN_02140	Beta-lactamase, class-A active site	Penicillin, Cephamycin, Cephalosporin
FGIJAEJN_00577	Tetracycline resistance protein TetA/multidrug resistance protein MdtG	Tetracycline
FGIJAEJN_00237	Tetracycline repressor TetR, C-terminal	Tetracycline
FGIJAEJN_04089	Tetracyclin repressor-like, C-terminal domain	Tetracycline
FGIJAEJN_01776, FGIJAEJN_03091, FGIJAEJN_04181, FGIJAEJN_02679, FGIJAEJN_00245, FGIJAEJN_02722, FGIJAEJN_02364, FGIJAEJN_00123, FGIJAEJN_02082, FGIJAEJN_02903, FGIJAEJN_00246, FGIJAEJN_02749	Tetracyclin repressor-like, C-terminal domain superfamily	Tetracycline
FGIJAEJN_03224	PsrA, tetracyclin repressor-like, C-terminal domain	Tetracycline

## Data Availability

Draft genome sequences and raw sequence datasets are deposited in NCBI Genbank, accessible through BioProject: PRJNA1073301.

## Supplementary Materials

The following supporting information can be downloaded at: https://www.mdpi.com/article/10.3390/microorganisms12040799/s1, Supplementary_Tables_Def.xlsx; table names: Table S1, sequencing, assembly, annotation and depth of coverage statistics; Table S2: details for Pannonibacter genome set; Table S3, JSpecies ANIblast result matrix; Table S4, JSpecies ANImummer result matrix; Table S5, JSpecies Tetranucleotide Correlation result matrix; Table S6, Tetranucleotide Correlation Search results for Pt1 genome; Table S7, Tetranucleotide Correlation Search results for Pt2 genome; Table S8, TYGS dDDH results for pairwise genome comparison; Table S9, ABRicate output for Pt1 and Pt2 genome assemblies; Table S10, InterPRO protein domain annotation on Pt1 genome assembly; Table S11, putative ARGs; Table S12, sequencing and assembly statistics for long-read sequencing (Pt2 sample); Table S13, Proksee VirSorter and Phigaro results for prophage sequence detection.
